# Human Luteinized Granulosa Cells—A Cellular Model for the Human Corpus Luteum

**DOI:** 10.3389/fendo.2019.00452

**Published:** 2019-07-09

**Authors:** Konstantin Bagnjuk, Artur Mayerhofer

**Affiliations:** Biomedical Center Munich (BMC), Cell Biology, Anatomy III, Ludwig-Maximilians-University (LMU), Planegg, Germany

**Keywords:** cell death, human ovary, paracrine action, extracellular matrix, hormone, proteomic, necroptosis

## Abstract

In the ovary, the corpus luteum (CL) forms a temporal structure. Luteinized mural granulosa cells (GCs), which stem from the ruptured follicle, are the main cells of the CL. They can be isolated from follicular fluid of woman undergoing *in vitro* fertilization. In culture, human GCs are viable for several days and produce progesterone, yet eventually steroid production stops and GCs with increasing time in culture undergo changes reminiscent of the ones observed during the demise of the CL *in vivo*. This short review summarizes the general use of human GCs as a model for the primate CL and some of the data from our lab, which indicate that viability, functionality, survival and death of GCs can be regulated by local signal molecules (e.g., oxytocin and PEDF) and the extracellular matrix (e.g., via the proteoglycan decorin). We further summarize studies, which identified autophagocytotic events in human GCs linked to the activation of an ion channel. More recent studies identified a form of regulated cell death, namely necroptosis. This form of cell death may, in addition to apoptosis, contribute to the demise of the human CL. We believe that human GCs are a unique window into the human CL. Studies employing these cells may lead to the identification of molecular events and novel targets, which may allow to interfere with CL functions.

## Introduction and Scope

To better understand the biology of human reproduction, studies addressing the human situation are required. This is due to distinct differences between humans and other mammalian species regarding structure and function of reproductive organs. The central organs of the female reproductive tract are the ovaries, which cannot be readily studied in the human.

The ovary is a highly dynamic organ that contains resting, growing, and dying follicles, as well as the corpus luteum (CL). During a menstrual cycle this temporal structure comes into existence upon ovulation and develops into an active, but short-lived (about 2 weeks) endocrine structure. It exists longer (months) if a pregnancy occurs, but in any case eventually it will shut down functionally and structurally. These events are termed functional and structural luteolysis.

Ovarian functions are frequently studied in animal models. In typical laboratory animals, such as mice and rats, the life time of the CL is restricted to only a couple of days, i.e., it is much shorter than in women. Domestic animals, while more similar to primates (1) with respect to the lifespan of the CL, differ in e.g., luteolytic signaling and the cellular events responsible for luteolysis (2). Therefore, non-human primates appear to be the best suited translational model for the human (3).

Due to the development of ART, a further possibility for the study of human ovary has opened. While connective tissue and newly formed blood vessel (4) are parts of the CL, its main cellular components are cells, which stem from the ovulatory follicle, in particular, mural luteinizing granulosa cells (GCs). These cells are by-products of follicular aspiration performed during IVF-procedures in women. They can be isolated, cultured, and studied.

As true for all cellular studies, cell culture phenomena must be considered and verification studies, involving human and/or non-human primate tissue are therefore required. If combined with such studies, we believe that cell cultures of human GCs are a unique window into the human ovary, and allow one to identify and study specifically events of relevance to the human CL.

This short review will briefly describe the cell culture model of human GCs, and will then summarize data mainly from our studies, in which we observed apoptosis and autophagocytotic events. We will then present recent data, which implicate necroptotic cell death in GCs and in human CL regression. We will here not discuss studies, which identified further factors of potential relevance to ovarian follicular fate, e.g., handling of catecholamines and generation of H_2_O_2_ (5–9).

## Human GC Culture—a Specific Window to the Human Corpus Luteum

As mentioned, human mural GC can be gathered during IVF procedures. Different methods have been developed to separate contaminating cells, namely leukocytes or erythrocytes, from granulosa cells. This can be achieved either by employing e.g., fluorescent activated cell sorting (FACS) and magnetic activated cell sorting (MACS, Dynabeads) or by physical properties (aggregation of GCs, density, selective adherence speed or size) (10). All methods except the so-called cell strainer technique involve a density gradient centrifugation step or a red blood cell lysis buffer (11) to eliminate specifically erythrocytes (12, 13). A comparison study came to the conclusion that the cell strainer method, developed by Kossowska-Tomaszczuk et al. (12), offers the best balance between purity, yield, speed, and simplicity (10, 12). We therefore employed this isolation method during the last years.

GCs stem from individual IVF-patients. While we have focused on cells derived from patients with male factor as the reason for IVF, the women undergoing this procedure are not a homogeneous population with respect of age, life style and medication. As well-known for all human subjects, heterogeneity between the derived GC samples is typically large. A usual consequence is that large number of repetitions are needed to obtain statistically valid results.

Most IVF patients undergo a controlled ovarian stimulation protocol (COS) before oocyte retrieval. Various COS have been established over time (14) and have distinct effects on the patients, their gonads and the outcome (birth rates) (15, 16). Most protocols include three main steps. First, drugs that interfere with gonadotropin releasing hormone (GnRH) are administered to shut down the natural pituitary-ovary axis. In a second step follicular stimulating hormone (FSH) is given. Prior to ovulation, oocyte maturation is forced by exogenously administered human chorionic gonadotropin (hCG), a luteinizing hormone (LH) analog. In primates the midcycle LH surge paves the way for *in vivo* luteinization of GCs (17, 18). Therefore, typical IVF-derived human GCs are most likely luteinizing or luteinized GCs and indeed they produce progesterone (19) like their *in situ*-counterparts. In case of pregnancy, hCG from the syncytiotrophoblast stimulates progesterone production and acts as a luteotrophic factor. Without hCG support the CL shuts down functionally and then, at least in some cases, it will regress to form a scar-like structure, the corpus albicans (20). How this is brought about in women is one of the many questions that remain to be studied.

To examine to what extend IVF-derived cultured GCs resemble their *in vivo*-counterparts, and are an appropriate model for the study of the CL (21), we combined a number of techniques, e.g., live cell imaging, cell death measurements, immunocytochemistry, and mass spectrometry analysis. We specifically studied GCs cultured from day 2 up to day 5 after retrieval from patients. The results were validated by comparison to *in vivo* generated non-human primate CL and immunohistochemical examinations of human and non-human primate ovary tissues.

*In vivo* follicular GCs differentiate upon ovulation to form large luteal cells (22). *In vitro*, the increase in size accompanying this differentiation is recapitulated and became likewise apparent over culture time. Large luteal cells express CYP11A1, a protein known to be crucial for progesterone production. Mass spectrometry revealed that it is highly expressed in IVF-derived human GCs (21, 23). Also low density lipoprotein receptor (LDL-R), 3β-hydroxysteroid dehydrogenase and steroidogenic acute regulatory protein (StAR) were found by mass spectrometry in cultured GCs. Next to CYP11A1 these proteins are indispensable for progesterone synthesis (24). In GCs, StAR, and LDL-R protein expression massively declined over culture time, which indicates a loss of steroidogenic function (21). *In situ*, the human corpus luteum only produces progesterone during a specific period of its lifetime (25). StAR expression is practically absent before the LH peak and the subsequent ovulation, is elevated during mid-phase CL and then is reduced in the regressing CL (26). Therefore, from an endocrine point of view, it appears that human, IVF-derived GCs are an apt model, especially for the study of the corpus luteum lifecycle from the mid-phase onwards.

Interestingly, Bildik et al. (15) recently showed that in comparison to naturally matured luteinized GCs, GCs from routine IVF-patients, who underwent COS, are more steroidogenic, express less anti-apoptotic but more pro-apoptotic factors and show a diminished viability under culture conditions. However, these characteristics vary and depend on the stimulation protocol (15). Thus, human, IVF-derived GCs may be specifically useful to study luteal phase defects, which sometimes occur during IVF procedures (27).

## A Sodium Channel, Oxytocin, PEDF, and Decorin Are Involved in GC Death

Over the years our studies with human GCs, isolated from follicular fluid of IVF patients, in combination with studies in human and non-human primate ovaries, led to the identification of a number of factors with the potential to influence the functions and the fate of these ovarian cells. Our studies addressed among others the role of a sodium ion channel, expressed by GCs, and the roles of oxytocin, PEDF, and decorin, i.e., secreted molecules, which may act in a paracrine fashion. We found that these molecules are intrinsically linked to different forms of cell death.

Human GCs, but also non-human primate luteal cells, express a sodium ion channel (*SCN9A*), which is regulated by the luteotrophic hormone hCG (28). Upon hCG-stimulation, *SCN9A* mRNA levels and ion channel activity were suppressed in GCs. The pharmacologically induced channel opening resulted in strikingly increased lysosomal activation and autophagocytic events. Autophagocytic events in the large granulosa luteal cells of the non-human primate CL were also deducted from results of electron microscopical examination and were later confirmed by further studies to occur in human CL (29). For instance, expression of the autophagy related protein Beclin-1 is elevated in the young CL, compared to old (regressing) CL, which may indicate that autophagy, in contrast to other cell death forms, may even be involved in maintaining the CL. This point requires further investigation, however. Recently, interconnectivity of different cell death forms was reviewed by Chen et al. (30). Although each cell death form has a distinct pathway some of the involved proteins act as molecular switches between different cell death pathways. Therefore, more than one cell death form can be activated simultaneously. In this context autophagy plays a special role as it can be seen as a mechanism with high potency to results in cell survival.

Apoptosis of luteal cells is known to be the main cause for the demise of the CL in rodents, but in humans it has been shown that only a small fraction of luteal cells undergoes apoptosis (20). Several factors may be involved in its initiation. Oxytocin (OT), a small peptide hormone was found in the human and non-human primate CL, as well as in the bovine ovary. It was hypothesized to act locally, e.g., as a luteolytic factor (31–33), because local production was accompanied by expression of the OT receptor (OTR) (34–36). Two studies in human mural GCs built on these results (37, 38) and confirmed expression of the receptor in GCs and CL. Exogenously applied to human GCs, OT acutely within seconds elevated the levels of intracellular Ca^2+^ and within hours, changed progesterone production (37). A further downstream role of OT in the induction of apoptosis of GCs became apparent in a follow-up study, also employing IVF-derived GCs. OT reduced viability by increasing caspase 3/7 activity, resulting in typical apoptosis, confirmed by electron microscopy (38). As OTR were found in the CL of humans and non-human primates, we concluded that the OT/OTR system may contribute to luteolysis.

Another factor identified more recently, is pigment-epithelium derived factor (PEDF). It is produced by GCs and was linked to anti-angiogenesis and apoptosis (39). We found that exogenously applied PEDF elevated reactive oxygen species (ROS) in human GCs, yet the exact mode of action of the multifunctional PEDF molecule and specifically the PEDF-receptor(s) involved, remain to be identified. PEDF and its ovarian roles are evolving. It has recently been shown to be involved in counteracting the activity of the hCG/vascular endothelial growth factor pathway and therefore may also attenuate ovarian hyperstimulation syndrome (OHSS) symptoms (40).

The CL is composed of the genuine follicle-derived cells (large and small luteal cells), blood vessels, connective tissue cells, and extracellular matrix. Extracellular matrix of the CL contains decorin (DCN), a proteogylcan, which was identified as a product of luteinized GCs (41). It is absent from the GC compartment of growing follicles in human and a non-human primate, but is present in the ovarian interstitial and theca compartments. It may leak from there to the follicular fluid, where it was observed in higher concentrations as in the blood serum (42). Interestingly it was recently suggested that its concentrations may be correlated with oocyte quality (42). DCN, besides its role in the “decoration” (hence the name decorin) and thereby the stabilization of collagen fibrils, also interferes with growth factor signaling (e.g., epidermal growth factor receptor; EGFR). DCN is also known to play a role in autophagocytotic events (43, 44), a possibility which however has not been examined in the ovary yet. Taken together, it is possible that DCN may be involved in the regulation of the life span of the CL via interactions with various ovarian and luteal growth factor systems. This assumption is based on the changing expression levels of DCN in the aging CL on one side, and the ability of DCN to reduce apoptosis (evidenced by caspase 3/7 activity) in GCs on the other side. This mode of action was, however, only found when GCs were studied that were cultured for 5 days, but not in “younger” cells. The results indicate that GCs *in vitro* i.e., during the time of culture change their behavior, an observation, which fostered the idea that studying such changes may be useful to identify events related to the fate of the CL.

## Identification of Necroptosis in GC and Luteal Cells

When we followed GC changes during culture time, we found that in addition to apoptosis, necroptosis takes place (45). Necroptosis is a form of regulated cell death, which involves a cascade of events and the formation of the necrosome, i.e., receptor interacting protein kinase 1, 3 (RIP1, RIP3) and phosphorylation and oligomerization of mixed lineage kinase domain like pseudokinase (MLKL), which is the executioner and unique marker for necroptosis (46, 47). Of note, blockers Of note, blockers of necroptosis [e.g., necrostatin 1 (Nec1) and necrosulfonamide (NSA)] have been developed, which prevent cell death. Necroptotic cells exhibit typical morphological signs of necrosis including ballooning and cell burst (21). In contrast to apoptosis, necro(pto)sis leads to porous cell membranes and, in the body, eventually to an immune reaction (48). Hence, necroptosis could be important for immune cell attraction to the CL, which appear to be crucial in its demise (20, 29, 49). Necroptosis in humans was until recently (21), described only in various pathologies, e.g., neurodegenerative disease, heart attack or brain injury (50). Our studies (21, 45, 51) and one in regressing bovine CL (52) extend this to physiological situations.

The reasons leading to necroptosis were described to vary significantly between different cells (47). In human cultured GCs necroptosis was observed under basal culture condition and like apoptosis progressed during culture time of GCs. It is possible that a small peptide (ARP), derived from a special splice form of acetylcholinesterase (AChE), namely AChE-R is involved. AChE-R and likely also ARP are formed by GCs and exogenous ARP, if added to cultured GCs, enhanced necroptosis.

The roles of acetylcholine (ACh) and AChE in the determination of life and death may go beyond the CL and include the GCs in the follicle, as concluded from studies in 3D cultured primate follicles (51). We found that elevation of ACh (induced by a blocker of AChE), as well as inhibition of necroptosis (using Nec-1) improved follicular growth. The results might be of translational relevance and may help to improve primate follicular growth *in vitro* and thus may allow to develop better strategies to preserve fertility in oncological patients.

In an attempt to examine underlying causes for basal cell death events in cultured GCs, we monitored cellular changes by employing mass spectrometry (21). The cellular proteins, which increased during a culture period of 5 days, belonged to distinct pathways, and results indicated that among others the ceramide salvage pathway is involved. It consists of enzymes including sphingomyelin phosphodiesterase 1 (SMPD1), acid ceramidase (ASAH1), galactosylceramidase (GALC), and glucosylceramidase beta (GBA) (53), all of which metabolize sphingolipids to generate ceramide and its metabolite sphingosine.

Sphingolipids consist of a sphingosine backbone linked to a fatty acid via an amide bond. Two metabolites, namely ceramide (CER) and sphingosine-1-phosphate (S1P) have excited researchers over many decades (54, 55). These two molecules are described to exert opposite actions on cell viability. For example, S1P blocks hydrogen peroxide induced apoptosis in primary GCs (56). In mice it was found that ceramide, on the other hand, induced cell cycle arrest and cell death in proliferating GCs (57). Further, this molecule was shown to induce apoptosis in human GCs, which could be rescued by prolactin (58). At the time of the mentioned study, Annexin-V staining was used as an apoptotic marker, however this kind of staining does not reliably distinguish between apoptosis and necroptosis, which was identified as a distinct form of cells death later. Of note, in the published pictures of the mentioned study, morphological signs for necrotic cell death are evident (e.g., ballooning).

Cell death of GCs in culture goes along with elevated LDH levels measured in the culture supernatant. This indicates that necro(pto)sis occurs. That necroptosis in GCs is linked to ceramides is based on the following experiments. We blocked endogenous ceramide production by fumonisin B1 (FB1) (59) and observed improved overall GC-viability. We also added exogenous ceramide and observed a reduction of viability. Results of blocker experiments using blockers of apoptosis (Z-VAD-FMK) or necroptosis (NSA, Nec-1s) finally identified that necroptosis is the principal form of cell death induced by endogenous ceramide. The presence of phosphorylated MLKL (pMLKL) in CL samples from different primate species and the appearance of pMLKL only in the regressing CL of non-human primate CL samples, support *in vivo* relevance, as do results of transcriptomic analysis of *in vivo* developed non-human primate CL (21).

## Summary

**Figure 1:**
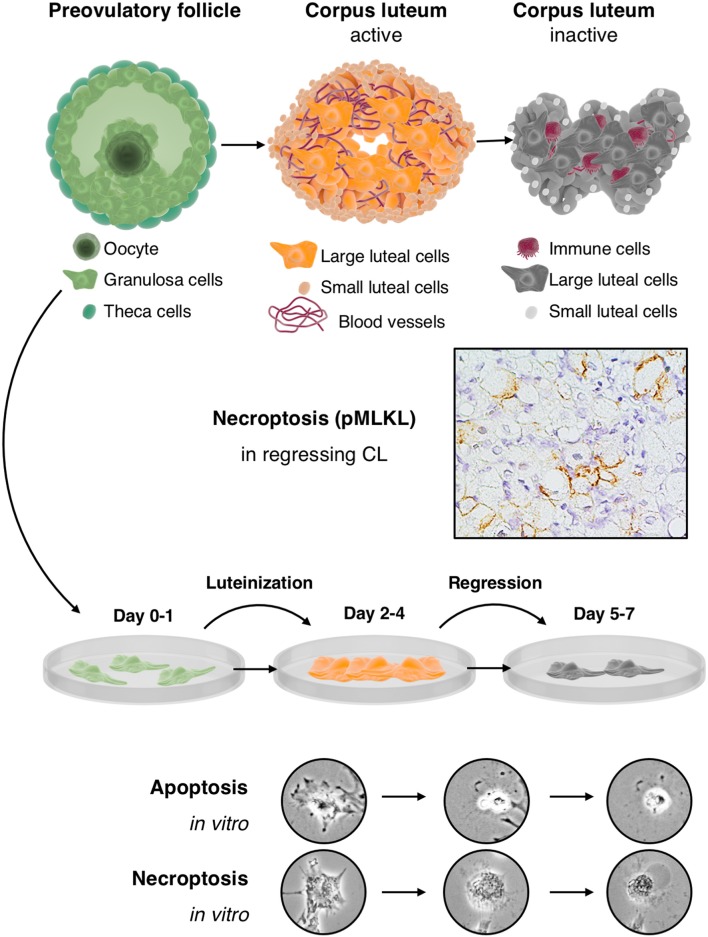
Cultured human GCs represent a model for the human CL, which comes into existence after ovulation and contains the GC-derived large luteal cells and other cell types: The upper part depicts the preovulatory follicle with its main cellular components, i.e., granulosa cells, the oocyte and theca cells (left), an active corpus luteum (middle), and a regressing corpus luteum (right). Mural GCs of the preovulatory follicle can be isolated and cultured from aspirated follicular fluid, which also contains cumulus cells and the oocyte. The active corpus luteum is a temporal organ, which is highly vascularized and produces progesterone. The inactive and regressing corpus luteum also contains immune cells. As shown in the second row, there is evidence for necroptosis in this structure, indicated by immunohistochemical detection of the necroptosis marker pMLKL in large luteal cells of a regressing corpus luteum (*M. mulatta*) (21). The third row schematically depicts isolated and cultured human GCs. This culture system recapitulates in a short period some of the main events occurring in the corpus luteum. In brief, isolated GCs luteinize in culture and produce progesterone but eventually die by apoptosis and necroptosis. These two cell death forms can be distinguished, albeit only if accompanied by other modes of detection (21), by morphological signs (two bottom rows). Apoptosis of GCs is typically characterized e.g., by a condensed cytoplasm and nucleus, whereas necroptotic cells show cellular ballooning.

IVF-derived cultured human GCs resemble luteinized large granulosa cells of the CL. They are a highly relevant *in vitro* model for the human CL, yet parallel studies in human or non-human primate tissues remain crucial because the CL also contains further cell types, which must be taken into consideration as players in the concert of luteal activity and regression. The data summarized in this review imply that during the first days of culture, GCs resemble the active CL, while at later time points they resemble the regressing CL (Figure 1). Studies employing these cells may lead to the identification of pathways, involved in the regulation of the CL. They may then pave the way for novel treatment strategies. For example, the recent identification of necroptosis suggest that blockers of necroptosis may be employed to possibly prevent luteolysis and may help to prevent luteal phase defects in ART patients.

## Author Contributions

AM conceived of the studies mentioned in the review. KB performed one of the most recent studies mentioned. KB and AM wrote the minireview and the figure. For generation of the figure, tools provided at https://www.somersault1824.com were used. This is gratefully acknowledged.

### Conflict of Interest Statement

The authors declare that the research was conducted in the absence of any commercial or financial relationships that could be construed as a potential conflict of interest.
